# Nomogram prediction of overall survival in breast cancer patients post-surgery: integrating SEER database and multi-center evidence from China

**DOI:** 10.3389/fonc.2024.1470515

**Published:** 2025-01-22

**Authors:** Yufen Zheng, Yuan Yuan, Minya Jin, Chunlong Wu

**Affiliations:** Department of Clinical Laboratory, Taizhou Hospital of Zhejiang Province Affiliated to Wenzhou Medical University, Linhai, China

**Keywords:** breast cancer, nomogram, overall survival, prognosis, SEER database

## Abstract

**Purpose:**

Overall survival (OS) in postoperative breast cancer patients is influenced by various clinicopathological features. Current prognostic methods, such as the 7th edition of AJCC staging, have limitations. This study aims to construct and validate a comprehensive nomogram integrating multiple clinicopathological features to predict OS more accurately in breast cancer patients.

**Methods:**

We identified 60,445 .female patients who underwent breast cancer surgery between January 1, 2011, and December 31, 2015, from the Surveillance, Epidemiology, and End Results (SEER) database, randomly divided into training and internal validation cohorts. Additionally, data from 332 breast cancer surgery patients from four hospitals in Taizhou, Zhejiang Province, were included as an external validation cohort. Kaplan-Meier analysis assessed the impact of clinicopathological features on OS, and multivariable Cox regression identified independent prognostic factors. A nomogram based on these factors was constructed to predict 1-, 3-, and 5-year OS. Model predictive performance was evaluated using C-index, AUC, calibration curves, and decision curves during internal and external validation.

**Results:**

Multivariable Cox regression analysis identified age, pathological grade, AJCC stage, ER status, PR status, and HER2 status as independent prognostic factors used in the nomogram construction. The nomogram achieved a C-index of 0.724 (95% CI, 0.716-0.732) in the training cohorts, 0.717 (95% CI, 0.705-0.729) in the internal validation cohorts, and 0.793 (95% CI, 0.724-0.862) in the external validation cohorts, indicating strong discriminative ability. Calibration curves demonstrated good agreement between predicted and observed outcomes in all validation cohorts. Decision curve analysis showed that the nomogram provided maximum net benefit across all validation cohorts.

**Conclusion:**

The nomogram developed in this study integrates multiple clinicopathological features and provides a convenient and accurate tool for predicting individualized OS in breast cancer patients. This tool can optimize treatment strategies and improve patient prognosis.

## Introduction

Breast cancer is among the most prevalent malignancies globally and ranks as the second leading cause of cancer-related deaths in women (1, 2). The incidence of breast cancer has notably increased in recent years due to changes in lifestyle and advancements in diagnostic technologies. Despite significant progress in treatment modalities, the exact etiology of breast cancer remains incompletely understood. Consequently, overall prognosis for breast cancer patients remains challenging.

The TNM staging system developed by the American Joint Committee on Cancer (AJCC) is widely recognized globally and utilized for predicting disease progression and guiding effective treatment strategies in breast cancer patients (3, 4). This system categorizes tumors based on tumor size (T), lymph node involvement (N), and distant metastasis (M). However, its applicability is significantly influenced by various clinicopathological features such as age, gender, histological grade, and breast cancer subtypes, all of which crucially impact patient survival outcomes (5, 6). Accurate prediction of postoperative prognosis in breast cancer patients is essential for devising personalized treatment plans aimed at improving survival rates in high-risk individuals and reducing mortality.

Nomograms are widely recognized as valuable and reliable clinical tools that integrate multiple clinical variables to assist clinicians and patients in estimating individual survival probabilities and formulating personalized treatment decisions (7, 8). Compared to traditional staging systems, nomograms offer significant advantages in terms of predictive accuracy and simplicity, thus emerging as a new standard for guiding cancer therapy (9).

This study aims to construct a detailed nomogram for predicting overall survival (OS) in postoperative breast cancer patients, specifically focusing on 1-year, 3-year, and 5-year survival probabilities. The training and internal validation cohorts for the nomogram were derived from the Surveillance, Epidemiology, and End Results (SEER) database of the National Cancer Institute, USA. To further validate the model’s accuracy and applicability, external validation was conducted using patient data from four hospitals in Taizhou, China. By integrating and analyzing a diverse range of clinical data, this study seeks not only to identify key factors influencing breast cancer prognosis but also to provide clinicians with a practical, accurate, and user-friendly tool for individualized survival prediction and risk assessment. This tool aims to optimize treatment decisions and improve survival outcomes for breast cancer patients.

## Methods

### Patients

The training and internal validation cohorts for this study were derived from the SEER (Version 8.3.8) database maintained by the National Cancer Institute, USA. Accessible at https://seer.cancer.gov/, this database spans data from 1975 to 2016 and was released for public use in April 2019 following its submission in November 2018. SEER encompasses detailed information on millions of cancer patients, including demographic data, tumor characteristics such as location, size, and morphology, diagnostic timelines, and follow-up survival status. The extensive coverage and comprehensive recording of SEER establish it as an indispensable data source in cancer research, facilitating robust analyses and insights into various aspects of cancer epidemiology, prognosis, and treatment outcomes.

Patient inclusion criteria from the SEER database include: (1) age ≥ 14 years at diagnosis; (2) primary site surgery for the tumor; (3) pathological diagnosis of breast cancer; (4) diagnosed between January 1, 2011, and December 31, 2015; (5) complete clinical, pathological, and follow-up data. Exclusion criteria were: (1) diagnosis based on cytology; (2) male patients; (3) missing key data including 7th edition AJCC stage, estrogen receptor (ER) status, progesterone receptor (PR) status, HER2 status, breast cancer subtype, survival time, and follow-up survival status. Ultimately, 60,445 eligible breast cancer patients were included from the SEER database, randomly divided into training (n=42,327) and internal validation (n=18,118) cohorts at a ratio of 7:3.

**Figure 1:**
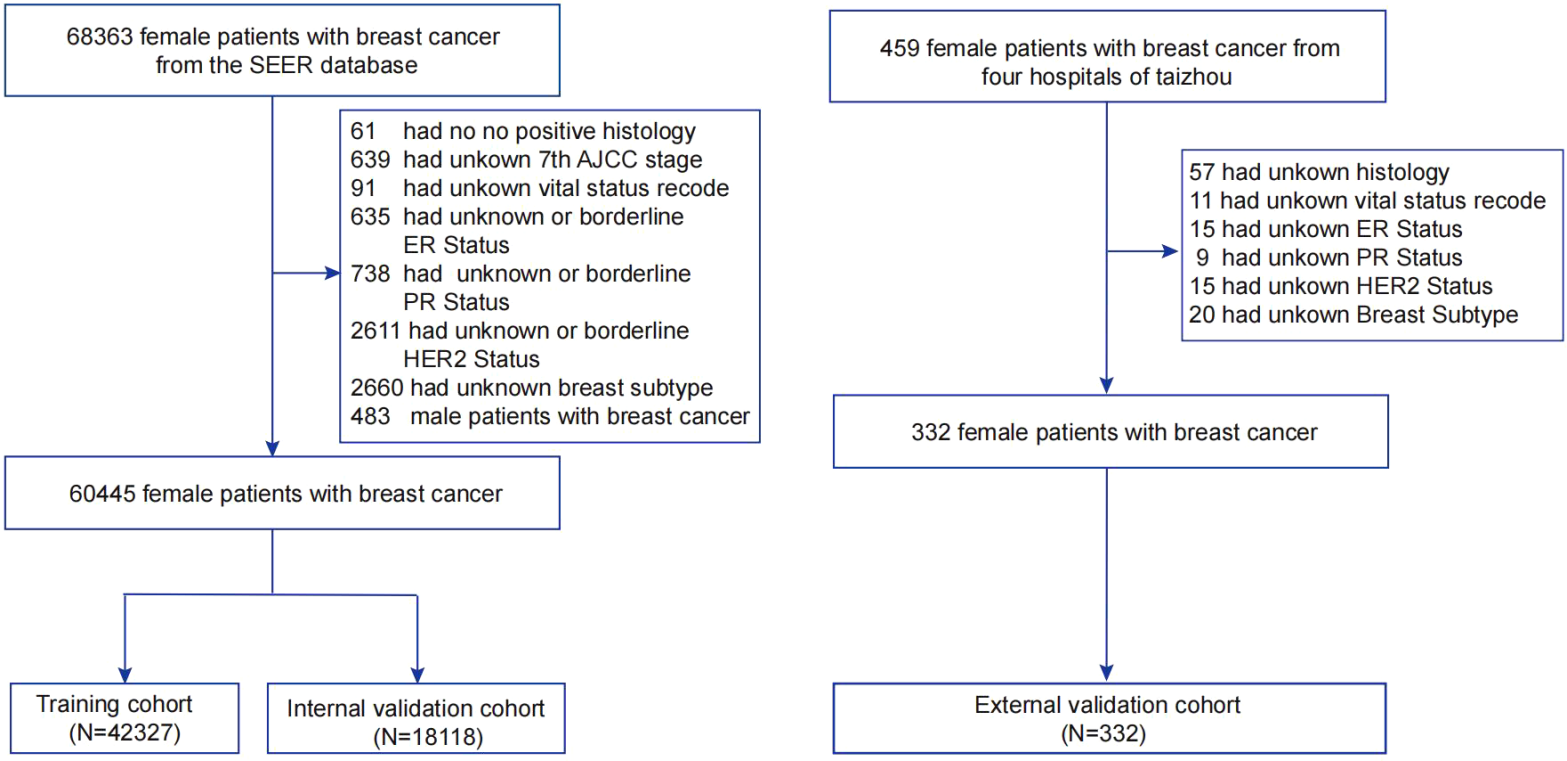
Flowchart of sample selection for this study. AJCC, American Joint Committee on Cancer; SEER, Surveillance, Epidemiology, and End Results; ER, estrogen receptor; PR, progesterone receptor; HER2, Human epidermal growth factor 2-neu.

To verify the model’s generalizability and robustness, data from four hospitals in Taizhou, Zhejiang Province, China, were collected as an external validation cohort. Inclusion and exclusion criteria mirrored those used for patients in the SEER database. Ultimately, 332 eligible breast cancer patients were included in the external validation cohorts. **Figure 1** illustrates the study workflow.

### Data acquisition

This study collected a range of clinicopathological data from breast cancer patients, including: age at diagnosis, tumor site, cancer grade (I, II, III, or IV), TNM staging according to the 7th edition of AJCC (I, II, III, or IV), tumor size (T0-T4), lymph node involvement (N0-N3), distant metastasis (M0-M1), ER status (negative, positive, or equivocal), PR status (negative, positive, or equivocal), HER2 status (negative, positive, or equivocal), molecular subtype, survival time, and follow-up survival status. TNM staging was defined according to the AJCC 7th edition guidelines (2010-2015). The follow-up cutoff for the external validation cohort was April 29, 2020, or the date of patient death, whichever came first. OS was defined as the time from diagnosis to death or last follow-up. Since data from the SEER database are publicly available, informed consent or ethical approval was not required. The external validation cohort of this study was approved by the Ethics Committee of Taizhou Hospital of Zhejiang Province (Project No.: K20210809).

### Statistical analyses

Categorical variables are presented as frequencies and percentages. Continuous variables are expressed as mean (standard deviation, SD) or median (interquartile range, IQR). Differences among the training, internal validation, and external validation cohorts for categorical variables were assessed using the chi-square test or Fisher’s exact test, while the t-test was used for normally distributed continuous variables and the Mann-Whitney U test for non-normally distributed continuous variables. Univariate and multivariate Cox proportional hazards regression models were employed to identify independent risk factors for OS in the training cohort. Kaplan-Meier curves were used to compare survival differences between different subgroups, and based on independent risk factors, a nomogram for breast cancer patients was constructed to predict 1-year, 3-year, and 5-year OS post-surgery. The predictive ability of the nomogram was validated using the internal and external validation cohorts. Calibration curves were used to compare predicted probabilities with observed frequencies; closer alignment with the 45-degree diagonal indicates better calibration (10). Harrell’s concordance index (C-index) and the area under the time-dependent receiver operating characteristic curve (AUC) were used to assess the accuracy of the nomogram (11). Decision curve analysis (DCA) evaluated the clinical utility and benefits of the predictive model, while clinical impact curves further validated the potential impact of the model in clinical practice (11). To ensure model stability and accuracy, we chose to exclude cases with missing data instead of using imputation methods, thereby reducing potential biases. All statistical analyses were performed using SPSS 22.0 and R software (version 4.0.3). A two-sided p-value less than 0.05 was considered statistically significant.

## Results

### Study population characteristics

**Table 1** summarizes the demographic and clinicopathological characteristics of breast cancer patients. A total of 60,777 patients were included in this study, comprising 42,327 in the training cohort, 18,118 in the internal validation cohort, and 332 in the external validation cohort. The median follow-up time across the cohorts was 49.0 months, during which 6,133 patients (10.09%) died. Among the patients, 34,544 (56.84%) were aged ≥60 years at diagnosis, 26,930 (44.31%) were classified as grade II in pathology, and over half (33,003 patients, 54.30%) were diagnosed with AJCC 7th edition stage I. Molecular subtypes included 6,590 (10.84%) patients with Triple-negative breast cancer, 15.81% were ER-negative, 26.49% were PR-negative, and 86.28% were HER2-negative.

**Table 1:** Clinicopathologic and baseline characteristics of breast cancer patients.

Variables	Total cohort (n=60777)	Training cohort (n=42327)	Internal validation cohort (n=18118)	External validation cohort (n=332)
Age, n (%)				
<60 years	26233 (43.16)	18236 (43.08)	7729 (42.66)	268 (80.72)
≥ 60 years	34544 (56.84)	24091 (56.92)	10389 (57.34)	64 (19.28)
Grade, n (%)				
I	15989 (26.31)	11068 (26.15)	4908 (27.09)	13 (3.92)
II	26930 (44.31)	18886 (44.62)	7880 (43.49)	164 (49.40)
III	17768 (29.23)	12315 (29.09)	5298 (29.24)	155 (46.69)
IV	90 (0.15)	58 (0.14)	32 (0.18)	0 (0.00)
7 th AJCC stage, n (%)				
I	33003 (54.30)	23112 (54.60)	9817 (54.18)	74 (22.29)
II	21665 (35.65)	15019 (35.48)	6445 (35.57)	201 (60.54)
III	5926 (9.75)	4069 (9.61)	1801 (9.94)	56 (16.87)
IV	183 (0.30)	127 (0.30)	55 (0.30)	1 (0.30)
ER, n (%)				
Negative	9610 (15.81)	6702 (15.83)	2822 (15.58)	86 (25.90)
Positive	51167 (84.19)	35625 (84.17)	15296 (84.42)	246 (74.10)
PR, n (%)				
Negative	16099 (26.49)	11188 (26.43)	4793 (26.45)	118 (35.54)
Positive	44678 (73.51)	31139 (73.57)	13325 (73.55)	214 (64.46)
HER2, n (%)				
Negative	52439 (86.28)	36630 (86.54)	15598 (86.09)	211 (63.55)
Positive	8338 (13.72)	5697 (13.46)	2520 (13.91)	121 (36.45)
Breast subtype, n (%)				
Luminal A	45817 (75.39)	32003 (75.61)	13674 (75.47)	140 (42.17)
Luminal B	5793 (9.53)	3965 (9.37)	1794 (9.90)	34 (10.24)
Her-2 enriched	2577 (4.24)	1732 (4.09)	726 (4.01)	119 (35.84)
Triple-negative	6590 (10.84)	4627 (10.93)	1924 (10.62)	39 (11.75)
Vital status, n (%)				
Alive	54644 (89.91)	38066 (89.93)	16288 (89.90)	290 (87.35)
Dead	6133 (10.09)	4261 (10.07)	1830 (10.10)	42 (12.65)
Median follow-up time (Months, 25th−75th percentile)	49.00 (34.00, 65.00)	49.00 (34.00,65.00)	48.00 (34.00,64.00)	71.00 (64.75,83.00)

AJCC, American Joint Committee on Cancer; ER, estrogen receptor; PR, progesterone receptor; HER2, Human epidermal growth factor 2-neu.

### Independent prognostic factors of OS

**Figure 2:**
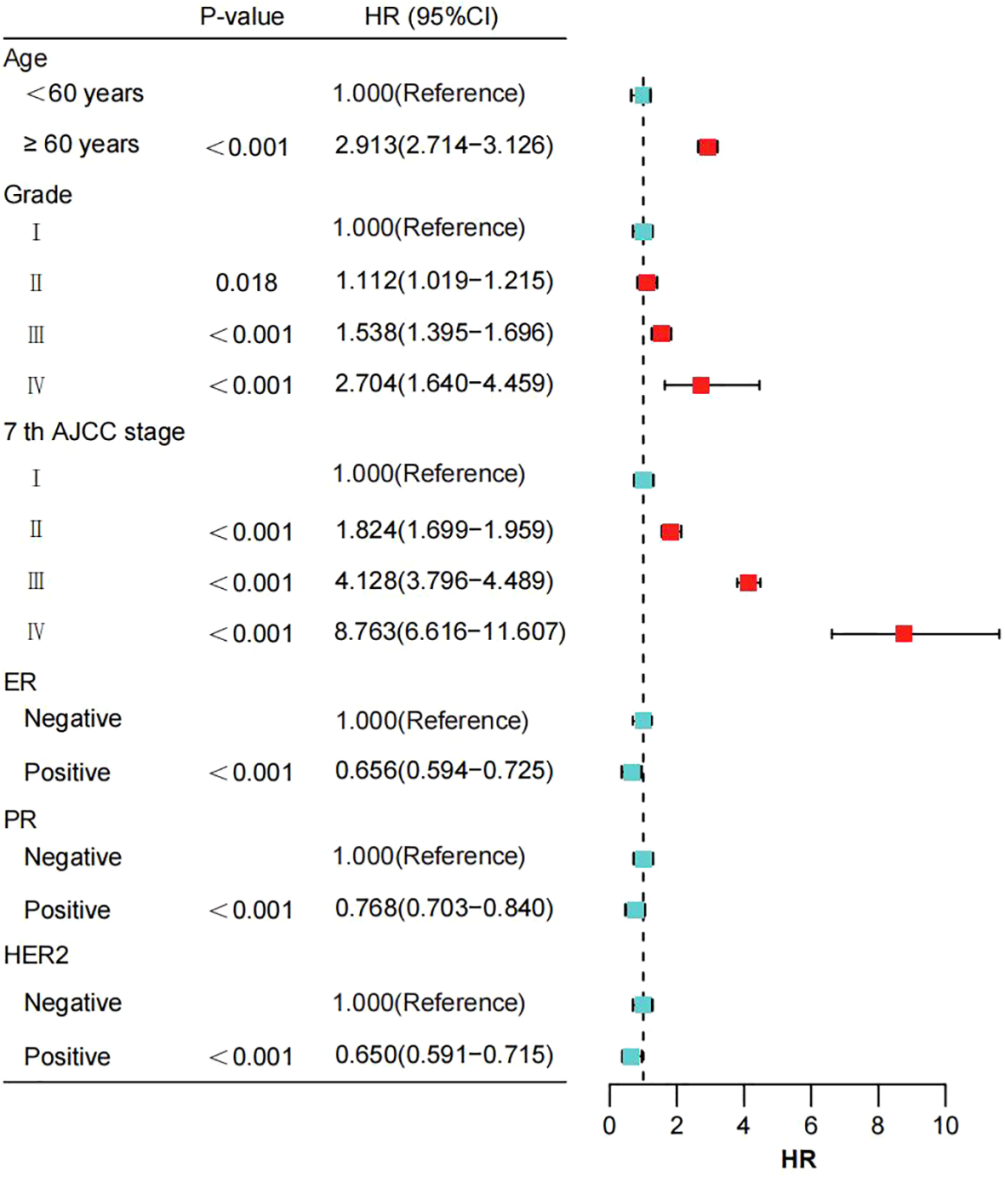
The impact of various prognostic factors in the training cohort is illustrated through a forest plot. AJCC, American Joint Committee on Cancer; ER, estrogen receptor; PR, progesterone receptor; HER2, Human epidermal growth factor 2-neu.

**Table 2** summarizes the results of univariate and multivariate Cox proportional hazards regression analyses in the training cohort of breast cancer patients. In the multivariate analysis, age, pathological grade, AJCC 7th edition stage, ER status, PR status, and HER2 status were identified as independent risk factors for OS (all P < 0.05). Compared to patients aged <60 years, those aged ≥60 years had a significantly increased risk of death with a hazard ratio (HR) of 2.913 (95% CI: 2.714 - 3.126, P < 0.001). Patients classified as grade II had a HR of 1.112 (95% CI: 1.019 - 1.215, P = 0.018), grade III had a HR of 1.538 (95% CI: 1.395 - 1.696, P < 0.001), and grade IV had a HR of 2.704 (95% CI: 1.640 - 4.459, P < 0.001) compared to grade I patients, indicating significantly increased risks of death. Notably, patients with grade IV had the highest risk of death, with a 2.704-fold increase compared to grade I patients. Similarly, compared to AJCC 7th edition stage I patients, those in stage II had a HR of 1.824 (95% CI: 1.699 - 1.959, P < 0.001), stage III had a HR of 4.128 (95% CI: 3.796 - 4.489, P < 0.001), and stage IV had a HR of 8.763 (95% CI: 6.616 - 11.607, P < 0.001), indicating significantly increased risks of death with advancing stages. Patients in stage IV had the highest risk of death, with an 8.763-fold increase compared to stage I patients. Furthermore, positivity for breast cancer molecular subtypes was associated with reduced risks of death. Specifically, ER-positive patients had a reduced risk by 0.656 (HR=0.656, 95% CI: 0.594 - 0.725, P < 0.001), PR-positive patients had a reduced risk by 0.768 (HR=0.768, 95% CI: 0.703 - 0.840, P < 0.001), and HER2-positive patients had a reduced risk by 0.650 (HR=0.650, 95% CI: 0.591 - 0.715, P < 0.001) (**Figure 2**).

**Table 2:** Univariate and multivariate COX proportional analysis of OS in the training cohort.

Variables	Univariate			multivariate		
	β	HR (95%CI)	P -Value	β	HR (95%CI)	P -Value
Age						
<60 years		1.000 (Reference)			1.000 (Reference)	
≥ 60 years	0.873	2.394 (2.234 - 2.567)	<0.001	1.069	2.913 (2.714 - 3.126)	<0.001
Grade						
I		1.000 (Reference)			1.000 (Reference)	
II	0.269	1.308 (1.200 - 1.426)	<0.001	0.106	1.112 (1.019 - 1.215)	0.018
III	0.838	2.311 (2.123 - 2.517)	<0.001	0.431	1.538 (1.395 - 1.696)	<0.001
IV	1.449	4.259 (2.595 - 6.988)	<0.001	0.995	2.704 (1.640 - 4.459)	<0.001
7 th AJCC stage						
I		1.000 (Reference)			1.000 (Reference)	
II	0.615	1.850 (1.727 - 1.983)	<0.001	0.601	1.824 (1.699 - 1.959)	<0.001
III	1.409	4.091 (3.773 - 4.435)	<0.001	1.418	4.128 (3.796 - 4.489)	<0.001
IV	2.134	8.452 (6.393 - 11.174)	<0.001	2.171	8.763 (6.616 - 11.607)	<0.001
ER						
Negative		1.000 (Reference)			1.000 (Reference)	
Positive	-0.811	0.444 (0.416 - 0.475)	<0.001	-0.422	0.656 (0.594 - 0.725)	<0.001
PR						
Negative		1.000 (Reference)			1.000 (Reference)	
Positive	-0.679	0.507 (0.477 - 0.539)	<0.001	-0.263	0.768 (0.703 - 0.840)	<0.001
HER2						
Negative		1.000 (Reference)			1.000 (Reference)	
Positive	-0.117	0.890 (0.812 - 0.975)	0.013	-0.430	0.650 (0.591 - 0.715)	<0.001

AJCC, American Joint Committee on Cancer; ER, estrogen receptor; PR, progesterone receptor; HER2, Human epidermal growth factor 2-neu; OS, overall survival.

**Figure 3:**
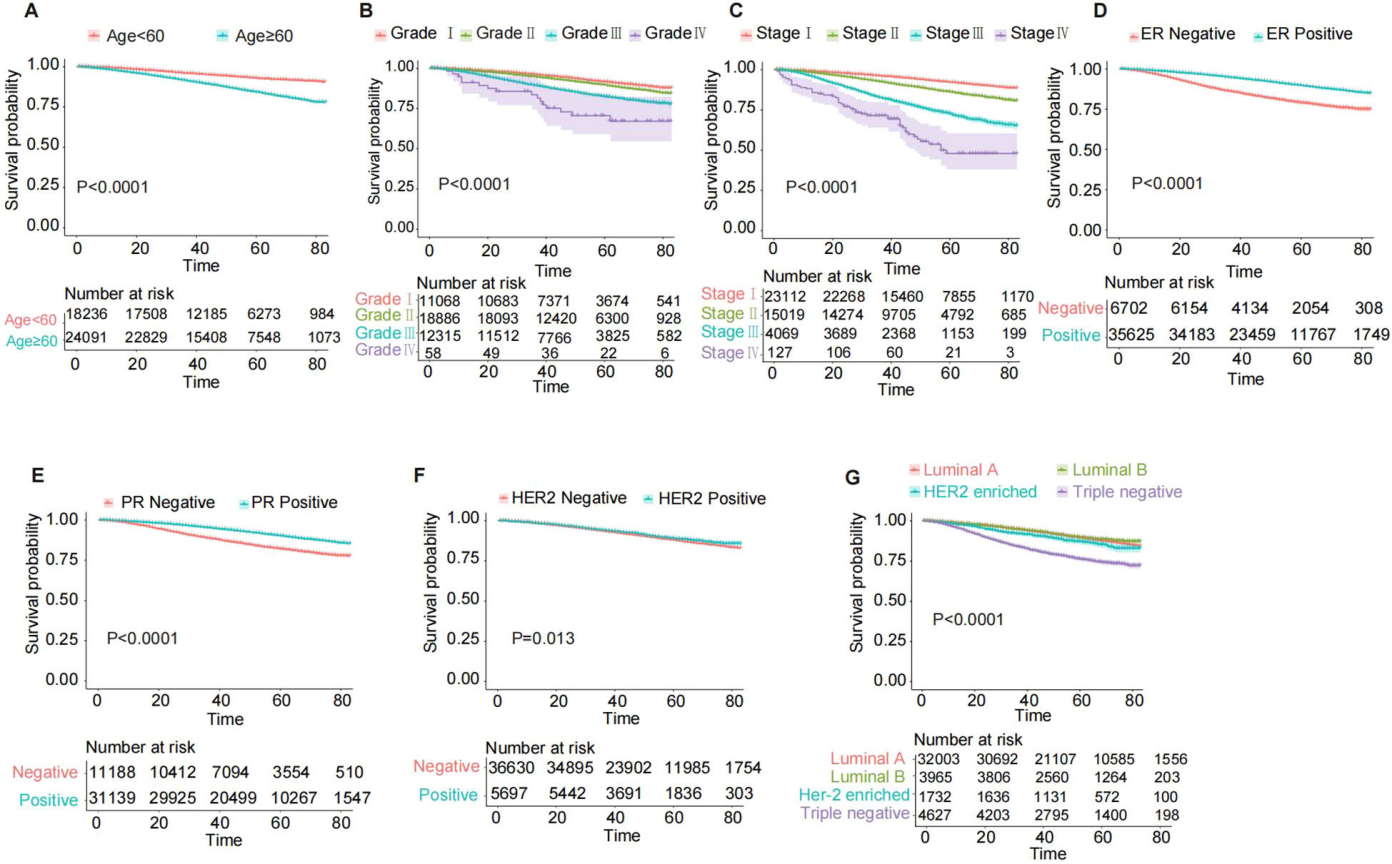
Kaplan-Meier OS curves for breast cancer patients in the training cohort, stratified by various characteristics. **(A)** Age; **(B)** Grade; **(C)** 7th AJCC stage; **(D)** ER status; **(E)** PR status; **(F)** HER2 status; **(G)** Breast subtype. AJCC, American Joint Committee on Cancer; ER, estrogen receptor; PR, progesterone receptor; HER2, Human epidermal growth factor 2-neu; OS, overall survival.

The Kaplan-Meier curves (**Figure 3**) demonstrate that breast cancer patients aged ≥60 years, ER-negative, PR-negative, HER2-negative, and those with triple-negative molecular subtype exhibited significantly lower overall survival rates (all P < 0.001). Additionally, patients classified as Grade IV and AJCC 7th edition stage IV had the poorest prognosis (all P < 0.0001).

### Nomogram construction

**Figure 4:**
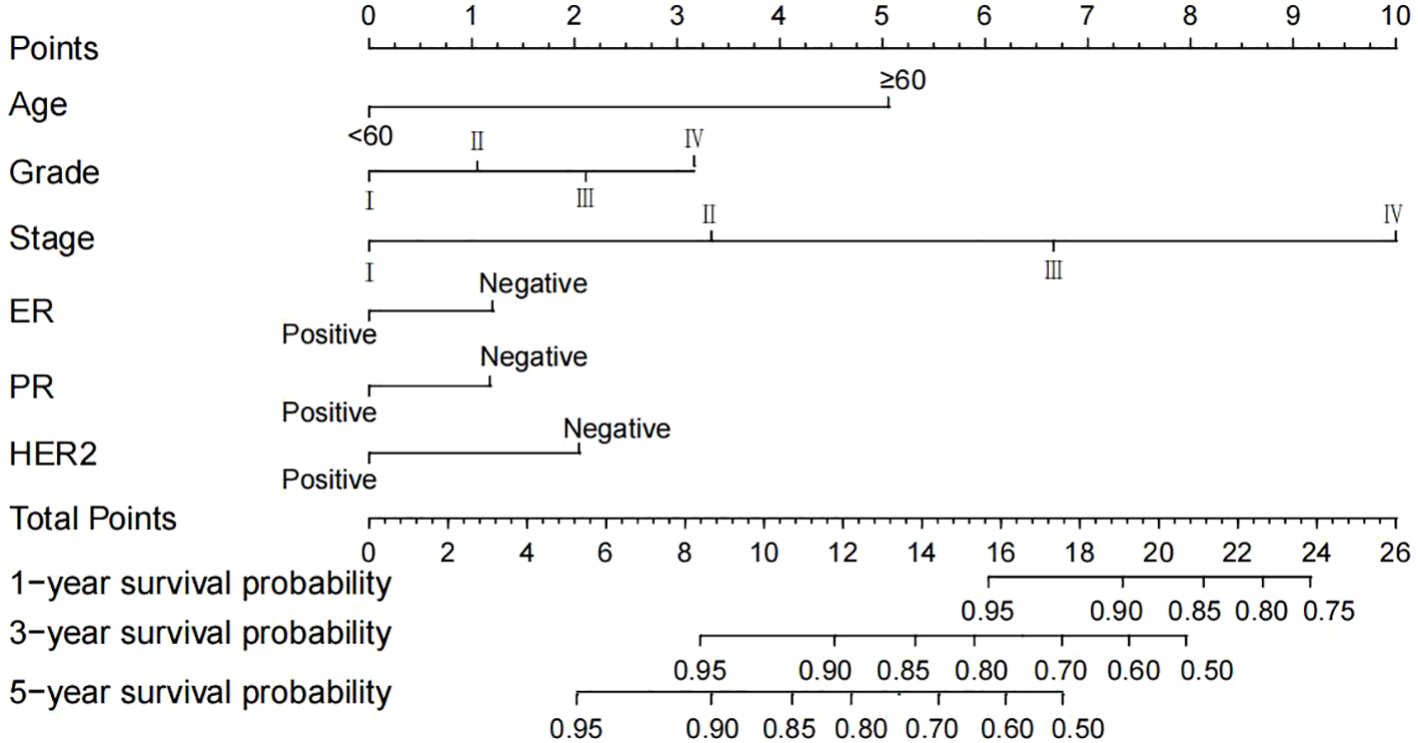
A nomogram predicting the 1-, 3- and 5-year OS of postoperative breast cancer patients in the training cohort. AJCC, American Joint Committee on Cancer; ER, estrogen receptor; PR, progesterone receptor; HER2, Human epidermal growth factor 2-neu; OS, overall survival.

Based on the multivariable Cox regression analysis, this study integrated age, histological grade, 7th edition AJCC stage, ER status, PR status, and HER2 status as independent prognostic factors to construct a nomogram predicting 1-, 3-, and 5-year OS rates for breast cancer patients (**Figure 4**). The nomogram illustrates that AJCC stage contributes most significantly to OS, followed by age and histological grade. For ease of use, each variable was assigned a score in the nomogram. To utilize the nomogram, one draws a vertical line upwards from the axis corresponding to each variable value to determine the score. These scores are then summed to locate the corresponding position on the total points axis, followed by drawing a line downward to the survival axis to estimate the probability of OS. For instance, a patient aged <60 years, with Grade III histological grade, Stage III AJCC, ER-negative, PR-negative, and HER2-positive status, would score 13.4. According to the nomogram, their estimated 1-, 3-, and 5-year OS probabilities are 95%, 87%, and 75%, respectively.

### Nomogram validation

**Figure 5:**
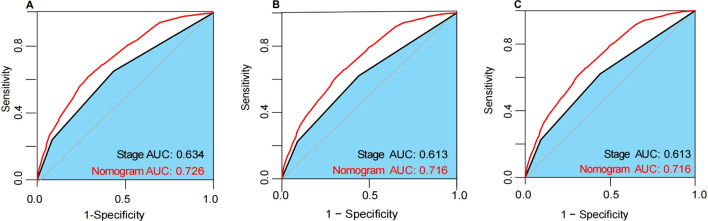
The ROC curves in the training cohort **(A)**, internal validation cohort **(B)** and external validation cohort **(C)**. AUC, area under the time-dependent receiver operating characteristics curve.

The nomogram demonstrated robust predictive ability across the training, internal validation, and external validation cohorts. In the training cohort, the nomogram achieved a C-index of 0.724 (95% CI, 0.716-0.732), in the internal validation cohort a C-index of 0.717 (95% CI, 0.705-0.729), and in the external validation cohort a C-index of 0.793 (95% CI, 0.724-0.862). These results indicate high discriminatory power and predictive accuracy of the nomogram. Furthermore, compared to the 7th edition AJCC staging system, the nomogram exhibited superior discriminative ability across all datasets. In the training cohort, the nomogram’s AUC was 0.726, whereas the AUC for the 7th edition AJCC staging was 0.634 (**Figure 5A**). In the internal validation cohort, the nomogram’s AUC was 0.716, compared to 0.613 for the 7th edition AJCC staging (**Figure 5B**). Similarly, in the external validation cohort, the nomogram’s AUC was 0.716, while the 7th edition AJCC staging had an AUC of 0.613 (**Figure 5C**). These findings further validate the nomogram’s superiority in prognostic assessment for breast cancer.

**Figure 6:**
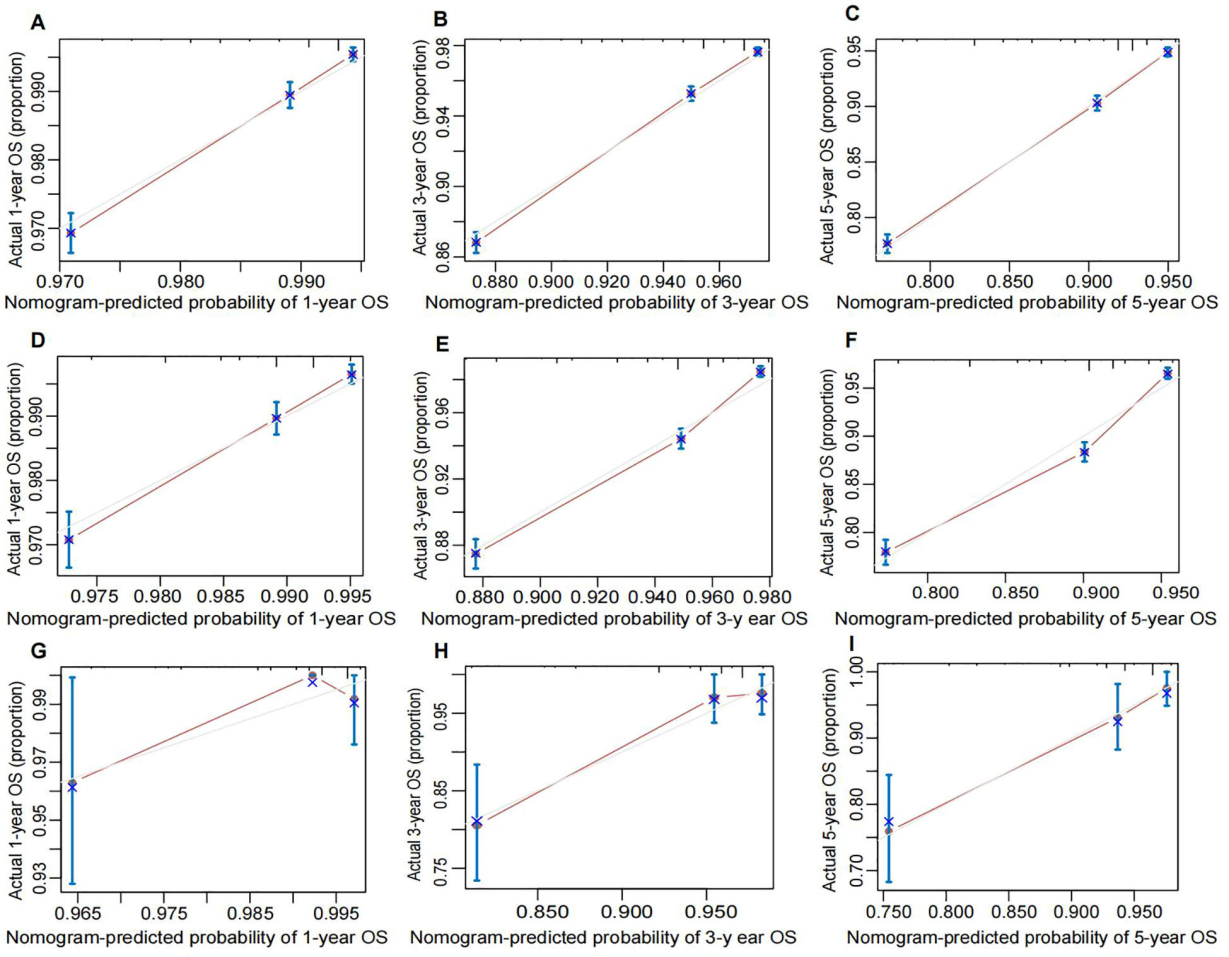
Calibration plot of the nomogram for predicting OS of breast cancer patients. **(A)** 1-year, **(B)** 3-year, and **(C)** 5-year OS in the training cohort. **(D)** 1-year, **(E)** 3-year, and **(F)** 5-year OS in the internal validation cohort. **(G)** 1-year, **(H)** 3-years, and **(I)** 5-years OS in the external validation coho cohort. The performance is estimated by bootstrap 1,000 repetitions. The X-axis plots the nomogram-predicted survival; the Y-axis plots the actual survival. OS, overall survival.

Calibration curves for predicting 1-year, 3-year, and 5-year OS in the training (**Figures 6A–C**), internal validation (**Figures 6D–F**), and external validation (**Figures 6G–I**)cohorts demonstrated excellent consistency between predicted probabilities and actual observed outcomes. These findings indicate high calibration accuracy of the nomogram across all datasets.

**Figure 7:**
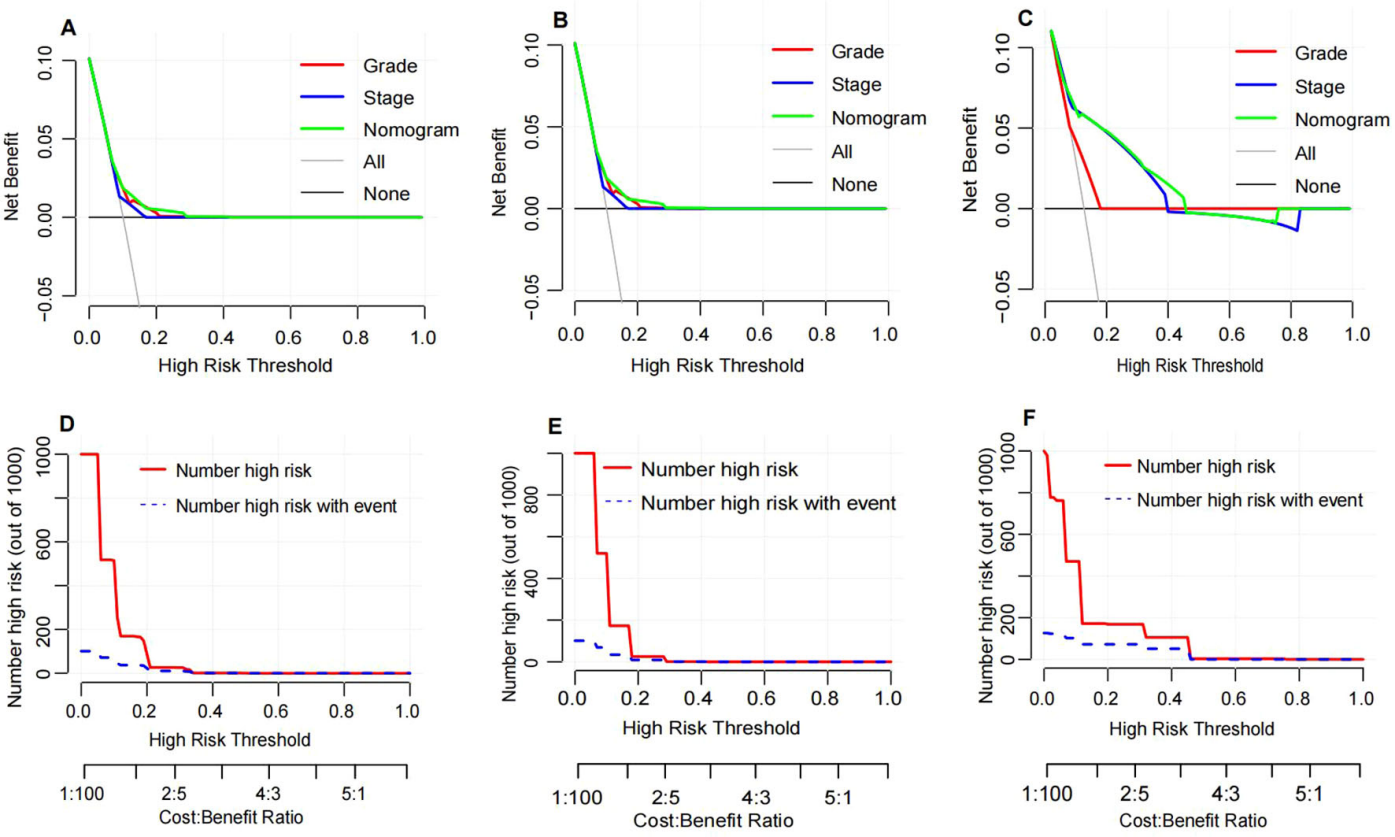
Decision curve analysis and clinical impact curve. Decision curve analysis for the nomogram in the training cohort **(A)**, internal validation cohort **(B)** and external validation cohort **(C)**. The horizontal solid black line represents the assumption that no patients will experience the event, and the solid gray line represents the assumption that all patients will relapse. Clinical impact curve of the nomogram for OS prediction in the training cohort **(D)**, internal validation cohort **(E)** and external validation cohort **(F)**.

Decision curve analysis (DCA) was conducted to assess the clinical utility of the nomogram and compare it with AJCC stage and pathological grade (**Figures 7A–C**). The DCA curves demonstrated that the nomogram provided the highest clinical net benefit in predicting OS probabilities across the training (**Figure 7D**), internal validation (**Figure 7E**), and external validation cohorts (**Figure 7F**). This indicates that the nomogram not only offers accurate personalized prognostication but also yields greater practical benefit in clinical decision-making.

## Discussion

In this study, we developed a nomogram integrating diagnostic age, pathological grade, AJCC 7th edition stage, ER status, PR status, and HER2 status to predict OS in postoperative breast cancer patients. The nomogram was validated in both internal and external validation cohorts, demonstrating superior predictive performance compared to the AJCC 7th edition stage (AUC: 0.735 vs 0.634). Calibration curves indicated high consistency between predicted and observed 1-year, 3-year, and 5-year OS rates across the training, internal validation, and external validation cohorts, highlighting the nomogram’s robust calibration ability for accurate survival prediction. Furthermore, DCA further underscored the clinical utility of the nomogram. DCA curves showed that the nomogram provided maximum net benefit across the training, internal validation, and external validation cohorts, significantly outperforming the AJCC 7th edition stage. This suggests that the nomogram not only enhances predictive accuracy but also offers substantial practical benefits in clinical decision-making. By facilitating the formulation of more personalized and effective treatment strategies, the nomogram has the potential to improve overall survival rates for breast cancer patients.

Based on the results of multivariable Cox regression analysis, we identified six clinical-pathological features as independent prognostic factors for OS in breast cancer patients, namely age at diagnosis, pathological grade, AJCC 7th edition stage, ER status, PR status, and HER2 status. The nomogram revealed that AJCC 7th edition stage was the most significant variable influencing OS, encompassing critical factors such as tumor size, lymph node involvement, and distant metastasis, all known to profoundly impact breast cancer prognosis (12, 13). Our findings highlight poorer prognosis among breast cancer patients aged over 60 years, consistent with prior research. Multiple studies have demonstrated a significant deterioration in prognosis with increasing age, underscoring a robust correlation between age and OS (14, 15). This phenomenon may be attributed to various factors, including clinical disease progression and age-related comorbidities (16). Moreover, aging may contribute to disease progression by impairing immune function and promoting tumor cell proliferation (17). Additionally, the nomogram indicated worse outcomes for breast cancer patients who tested negative for ER and PR, aligning with previous research findings (18, 19). Poorly differentiated tumors and triple-negative breast cancer are recognized as adverse prognostic indicators in breast cancer patients, findings corroborated by our study’s results (15, 20).

Nomograms serve as graphical tools for statistical prediction models, offering intuitive representations of specific outcome probabilities (21–23). They are characterized by parameters that are easy to acquire and measurable. Nomograms demonstrate excellent discriminative ability and exhibit high consistency between predicted and observed outcomes. In prognostic assessment, their economic and practical utility has been well-established across various tumor types. For instance, Kong et al. (6) developed a nomogram integrating age, tumor size, differentiation grade, N stage, M stage, and tumor location to accurately predict OS in patients with gastroenteropancreatic neuroendocrine tumors. Similarly, Kong et al. (6) integrated age at diagnosis, T stage, N stage, and M stage to construct a reliable and effective nomogram for predicting prognosis in patients with postoperative adrenocortical carcinoma.

Increasing evidence across various tumor types suggests that nomograms, compared to traditional AJCC TNM staging, possess superior predictive capabilities (23–25). In contrast to the widely used AJCC TNM staging system, our nomogram not only offers straightforward operation but also provides precise individualized prognostic assessments for diverse patient populations. Therefore, nomograms will aid clinicians in more accurately estimating individual patient survival probabilities and optimizing treatment strategies, thereby achieving enhanced clinical outcomes.

In addition to the reliable data source for our nomogram, our study presents several notable strengths. Firstly, we utilized a rich and comprehensive dataset of clinical and pathological characteristics from the SEER database, laying a robust foundation for constructing an accurate and reliable prognostic nomogram. Secondly, the nomogram demonstrates superior discriminative ability in predicting OS compared to the 7th edition AJCC staging system, validated comprehensively through internal and external validation cohorts. Lastly, our study incorporates six clinical and pathological variables, widely accessible and utilized in clinical practice, ensuring the nomogram’s simplicity and usability as a practical tool for clinicians to optimize treatment strategies and improve patient outcomes.

Despite the numerous strengths of this study, several limitations warrant consideration. Firstly, as this is a retrospective study based on the SEER database, inherent selection bias is challenging to fully mitigate. Therefore, future studies should consider cross-referencing with other databases or tools to validate findings in subsamples, thereby enhancing the model’s generalizability and reducing potential bias. Secondly, the SEER database has limitations in providing comprehensive clinical information, particularly regarding endocrine therapy, targeted therapies, and laboratory data. This lack of detailed clinical data may constrain the sensitivity and specificity of our survival prognostic model. The absence of such clinical data could impact the model’s performance and applicability to diverse patient populations. Thirdly, excluding cases with missing data may have reduced the sample size, potentially affecting the discriminative and predictive abilities of the survival prognostic model. To address this limitation, future studies should aim for more comprehensive data collection and consider employing prospective study designs to further validate and enhance the model’s stability and clinical utility. Finally, prospective studies have clear advantages in providing higher-quality and more comprehensive clinical data, allowing for more reliable causal inference and better capture of long-term efficacy and patient outcomes. Future research should actively explore prospective designs to complement and validate the findings of this study.

## Conclusions

This study introduces a nomogram for predicting postoperative OS in breast cancer patients. The nomogram, characterized by its simplicity and user-friendliness, facilitates personalized risk assessment and survival prediction effectively. With its accuracy and efficiency, this tool provides clinicians with personalized consultation, timely monitoring, and comprehensive clinical assessment, thereby optimizing treatment strategies and improving patient prognosis. Constructed based on rich and comprehensive clinical-pathological data, the nomogram has been validated through internal and external validation cohorts, demonstrating its reliability and practicality in clinical applications. This underscores its substantial potential to enhance clinical decision-making and patient outcomes.

## Data Availability

The datasets used and analyzed during the current study are available from the corresponding author on reasonable request.
